# Supplementary material for: High-Throughput Sequencing Reveals the Mycoviral Diversity of the Pathogenic Grape Fungus Penicillium astrolabium During Postharvest
Source: Viruses. 2025 Jul 28;17(8):1053. doi: 10.3390/v17081053 (PMC12390298; doi:10.3390/v17081053)
Supplement: Supplementary file 1 [file viruses-17-01053-s001.zip › viruses-3716777-supplementary.pdf]

Tabel S1 Sequence information of identified novel mycoviruses

Name of putative viruses	RdRP length (bp)	Best match	Identity (%)	Genome type	Family	Host
Penicillium astrolabium chrysovirus 1 (PaCV1)	3709	Penicillium janczewskii chrysovirus 1	83.48	dsRNA	*Chrysoviridae*	WHG8
Penicillium astrolabium partitivirus 1′ (PaPV1′)	1876	Diplodia seriata partitivirus 1	63.41	dsRNA	*Partitiviridae*	All strains
Penicillium astrolabium partitivirus 2 (PaPV2)	1895	Penicillium aurantiogriseum partitivirus 1	86.57	dsRNA	*Partitiviridae*	All strains
Penicillium astrolabium partitivirus 3 (PaPV3)	1752	Penicillium stoloniferum virus F	99.26	dsRNA	*Partitiviridae*	All strains
Penicillium astrolabium narnavirus 1 RNA1 (PaNV1 RNA1)	2512	Oidiodendron maius splipalmivirus 1 RNA1	73.99	+ssRNA	*Narnaviridae*	All strains
Penicillium astrolabium narnavirus 1 RNA2 (PaNV1 RNA2)	2522	Oidiodendron maius splipalmivirus 1 RNA2	74.74	+ssRNA	*Narnaviridae*	All strains
Penicillium astrolabium alphaflexivirus 1 (PaAFV1)	3341	Narcissus mosaic virus	99.12	+ssRNA	*Alphaflexiviridae*	WHG3-3, WHG8, WHG9, WHG10, and WHG11

Tabel S2 Novel mycoviruses sequence with partial missing information

Name of putative viruses	contig counting	Best match	Family	Genome type	Host
Penicillium astrolabium picobirnavirus 1(PaPBV1)	1	Lysoka partiti-like virus	*Picobirnaviridae*	dsRNA	WG6
Penicillium astrolabium picobirnavirus 2(PaPBV2)	3	Guyuan tick virus 1	*Peribunyaviridae*	-ssRNA	WG6
Penicillium astrolabium discovirus 1(PaDV1)	5	Penicillium discovirus	*Discoviridae*	-ssRNA	WG6
Penicillium astrolabium discovirus 2(PaDV2)	2	Penicillium roseopurpureum negative ssRNA virus 1	*Discoviridae*	-ssRNA	WG6
Penicillium astrolabium botourmiaviridae(PaBV)	2	Tongren Botou tick virus 1<br>Tongren Botou tick virus 1	*Botourmiaviridae*	+ssRNA	WG9
